# Leonurine Attenuates Obesity-Related Vascular Dysfunction and Inflammation

**DOI:** 10.3390/antiox11071338

**Published:** 2022-07-08

**Authors:** Xiao-Dong Shi, Jia-Xin Zhang, Xi-De Hu, Tao Zhuang, Ning Lu, Cheng-Chao Ruan

**Affiliations:** 1 Department of Physiology and Pathophysiology, School of Basic Medical Sciences, Fudan University, Shanghai 200032, China; 20211010004@fudan.edu.cn (X.-D.S.); huxide@stu.xzhmu.edu.cn (X.-D.H.); zhuangtao_@fudan.edu.cn (T.Z.); 2 Department of Clinical Medicine, Shanghai Medical College, Fudan University, Shanghai 200032, China; 18301050240@fudan.edu.cn

**Keywords:** obesity, vascular dysfunction, inflammation, oxidative stress, LEO, YTHDF1

## Abstract

Oxidative stress in adipose tissue is a crucial pathogenic mechanism of obesity-associated cardiovascular diseases. Chronic low-grade inflammation caused by obesity increases ROS production and dysregulation of adipocytokines. Leonurine (LEO) is an active alkaloid extracted from Herba Leonuri and plays a protective role in the cardiovascular system. The present study tested whether LEO alleviates inflammation and oxidative stress, and improves vascular function in an obese mouse model. Here, we found that obesity leads to inflammation and oxidative stress in epididymal white adipose tissue (EWAT), as well as vascular dysfunction. LEO significantly improved inflammation and oxidative stress both in vivo and in vitro. Obesity-induced vascular dysfunction was also improved by LEO as evidenced by the ameliorated vascular tone and decreased mesenteric artery fibrosis. Using mass spectrometry, we identified YTHDF1 as the direct target of LEO. Taken together, we demonstrated that LEO improves oxidative stress and vascular remodeling induced by obesity and targets YTHDF1, raising the possibility of LEO treating other obesity-related metabolic syndromes.

## 1. Introduction

Obesity originates from an imbalance between caloric intake and energy expenditure that promotes excessive fat storage and being overweight. The principal characteristic is that the chronic low activation of the immune system leads to the body showing a chronic low-grade inflammatory state [1,2]. Adipocytes and immune cells secrete a variety of adipokines including hormones, cytokines, and ROS, which influence the function of nearby or distant organs and tissues through autocrine, paracrine, or endocrine mechanisms [3]. Abnormal adipokine secretion in obese people leads to hypertension, atherosclerosis, and other cardiovascular diseases [4].

Oxidative stress induced by obesity has also been reported in the regulation of obesity-related cardiovascular disorders [5]. The activity of antioxidant enzymes, such as SOD (superoxide dismutase), CAT (catalase), and GPx (glutathione peroxidase), is significantly reduced, while that of nicotinamide adenine dinucleotide phosphate oxidase (NOX) is enhanced as adipose tissue is increased [6,7]. NOX4 is the major NOX isoform in adipocytes and ROS production in early obesity depends on NOX4 transferring electrons from NADPH to oxygen [8]. Another potentially significant source of ROS in the intermediate stages of obesity is activation of NOX2 by macrophages and immune cells [9]. Inflammation is another important source of oxidative stress in obesity [10]. TNF-α (tumor necrosis factor-α) and IL-6 (interleukin-6), as the most well-known mediators of the early inflammatory response, increase the activities of NOX and the production of superoxide anion [11]. 

Herba Leonuri, the herbaceous flowering plant, can be used for the treatment of uterine bleeding, uterine atresia, dystocia, and other gynecological diseases according to the compendium of Ben Cao Gang Mu [12]. LEO, a main alkaloid from Herba Leonuri, has been shown to have anti-platelet aggregation and contractile effects on the uterus. By modifying the structure of LEO, the shortcomings regarding the low bioavailability, weak transmembrane ability, and poor fat solubility of LEO have been solved [13]. Previous studies have shown that LEO has a protective effect on the cardiovascular system, such as providing protection against hypoxia-induced myocyte cell death and myocardial infarction, attenuating myocardial fibrosis and preventing atherosclerosis [14]. Importantly, LEO has been proven to have anti-inflammatory and antioxidant activity. LEO inhibits COX-2 (cyclooxygenase-2) mRNA and protein expression in LPS-induced mouse mastitis [15]. In addition, LEO can ameliorate the oxidative stress damage and insufficient angiogenesis of HUVECs induced by H_2_O_2_ [16]. In the present study, we aimed to examine whether LEO alleviates obesity-induced inflammation and oxidative stress, and improves vascular function in vivo and in vitro. Furthermore, we identified that the potential direct target of LEO is YTHDF1. Our study may lead to a potential novel therapeutic strategy for obesity-related vascular dysfunction.

## 2. Materials and Methods

### 2.1. Chemicals and Materials

Reagents used in this study were analytic-grade reagents provided by commercial suppliers; relevant information is in Table S1.

### 2.2. Animal and Diets

Male C57BL/6J mice aged 8 weeks were obtained from the Charles River company housed in a pathogen-free facility under a 12 h light/12 h dark cycle at a controlled room temperature of 22–25 °C and a relative humidity of 40–60%, with free access to water and food (if not specified). The mice were divided into three groups including chow, high-fat diet (HFD), and high-fat diet + LEO (HFD + LEO). Chow provided 10% of calories from fat (SLACOM, China). High-fat diet (HFD) provided 60% of calories from fat (SLACOM, China). LEO (40 mg/kg) or PBS was administered once daily for 12 weeks.

### 2.3. Anatomic Assessment and Biochemical Analyses

The adipose tissue, great vessels, and mesenteric artery were removed at the end of model. The adipose tissue, including SWAT (subcutaneous white adipose tissue), EWAT (epididymal white adipose tissue), and BAT (brown adipose tissue), were weighed. Mouse serum was collected and then total cholesterol and total triglycerides were measured by the automatic biochemical analyzer (C311, Roche). 

### 2.4. Quantitative Real-Time PCR

Total RNA of the adipose tissues and cells was extracted with the RNA Purification Kit and reversely transcribed to cDNA using the RT Master Mix. qPCR was performed using the SYBR Green Master Mix according to the manufacturer’s instructions. Data were normalized to the housekeeping gene (mouse GAPDH). Primers are listed in Table 1.

### 2.5. Haematoxylin and Eosin Staining

The SWAT, EWAT, BAT, liver, and mesenteric artery of the chow, high-fat diet (HFD), and high-fat diet + LEO (HFD + LEO) group were fixed in 4% paraformaldehyde; dehydrated in 30%, 50%, 75%, and 100% of the alcohol; embedded in paraffin; and sectioned. The EWAT of these groups was stained with hematoxylin and eosin. H&E staining images were obtained using an optical microscope. ImageJ was used to quantify the fat cell size.

### 2.6. Oil Red O Staining

The IWAT, BAT, EWAT, liver, and mesenteric artery of the chow, high-fat diet (HFD), and high-fat diet with LEO (HFD + LEO) group were fixed in 4% paraformaldehyde; dehydrated in 10%, 20%, and 30% sucrose solution; embedded in OCT; and sectioned. The sections of the liver were returned to room temperature, stained with oil red dye for 30 min, and differentiated with isopropyl alcohol for 20 s. Finally, the sections were washed by water and sealed with glycerin gelatin seal tablets. ImageJ was used to quantify the positive area and signal strength.

### 2.7. Immunofluorescence

The EWAT sections from three groups of mice were labeled with anti-F4/80 (1:100) overnight at 4 °C and then incubated with a mixture of Alexa Fluor 488-conjugated secondary antibodies for 1 h. Nuclei of the sections were counterstained with DAPI.

### 2.8. Masson’s Trichrome Staining

The primary branch of the mesenteric artery was isolated and paraffin-embedded. Collagen content was measured by Masson’s trichrome staining, with blue areas representing collagen fibers. The percentage of collagen fibers was quantified as the area of blue staining divided by the total area of mesenteric arteries. An optical microscope was used to obtain the images. ImageJ was used to quantify the positive area ratio.

### 2.9. In Vitro Mouse Mesenteric Artery Ring Experiment

After cervical vertebrae removal and death, the main mesenteric artery was separated and immediately placed in pre-cooled (4 °C) K-H solution. After removing the peripheral connective tissue and separating the surrounding fat, the blood vessels were cut into 3~4 mm long arterial rings, which were carefully pierced on two stainless steel hooks and then hung in a bath dish filled with K-H liquid. The lower steel wire of the vascular ring was fixed with a stainless steel hook and the upper end was connected with a tension transducer. O_2_ was continuously penetrated into the bath dish and 3~5 bubbles per second was appropriate. A constant-temperature water tank was connected to maintain the constant temperature at (37 ± 0.5) °C and a tension transducer was connected to a multichannel physiological recorder. After adjusting the resting tension of the mesenteric artery ring to 500 mg, K-H solution was replaced every 15 min for balancing. After balancing for 1 h, Phe (1 × 10^−6^ mol/L) was added into the bath dish for pre-contraction. Follow-up tests were started after elution to baseline. The final concentrations of ACH in the bath were 1 × 10^−9^, 3 × 10^−9^, 1 × 10^−8^, 3 × 10^−8^, 1 × 10^−7^, 3 × 10^−^^7^, 1 × 10^−^^6^, 3 × 10^−6^, and 1 × 10^−5^ mol/L, and the vasodilation amplitude was recorded. After the arterial rings were balanced, Phe (1 × 10^−6^ mol/L) was added into the bath dish to shrink the arterial rings to baseline. The final concentrations of SNP in the bath dish were 1 × 10^−9^, 3 × 10^−9^, 1 × 10^−8^, 3 × 10^−8^, 1 × 10^−7^, 3 × 10^−7^, 1 × 10^−6^, 3 × 10^−6^, and 1 × 10^−5^ mol/L, and the vasodilation amplitude was recorded.

### 2.10. Cell Culture

The RAW264.7 cell line was from Cell Bank of Chinese Academy of Sciences and maintained in 1640 medium containing L-glutamine, phenol red, and NaHCO_3_. Raw264.7 was pretreated with or without 20 μM LEO for 3 h and followed by TNF-α (100 ng/mL) for 12 h. RAW264.7 was grown to confluence in 60 mm dishes for western blot experiments and 12-well plate dish for quantitative real-time PCR analysis.

### 2.11. Pull-Down Assay and Western Blot

After RAW264.7 macrophages were treated with or without biotin-LEO for 6 h, proteins in the RAW264.7 macrophages were extracted. Total proteins were incubated with streptavidin beads for 2 h at 4 °C and then beads were washed three times with the lysis buffer. After resuspending beads, protein extracts were subjected to electrophoresis in SDS-polyacrylamide gels and transferred to the PVDF membrane. The membranes were blocked with fat-free dry milk for 1 h and incubated primary antibody overnight at 4 °C. The membrane was washed with TBST and the secondary antibody was incubated for 1 h. Immunopositive bands were shown after incubation with enhanced chemiluminescence reagents.

### 2.12. Statistical Analyses

All data are presented as means ± SEM. In all analyses, *p* < 0.05 was taken to indicate statistical significance. The data is normally distributed. Differences in mean values between two groups were assessed using the two-tailed Student’s t-test. Differences in mean values among more than two groups were determined using analysis of variance (ANOVA). If results from one-way ANOVA were significant, pair-wise differences between groups were estimated using Tukey’s post hoc test. GraphPad Prism 8 was used to analyze the data.

## 3. Results

### 3.1. LEO Alleviates Obesity and Lipid Deposition Induced by HFD

**Figure 1:**
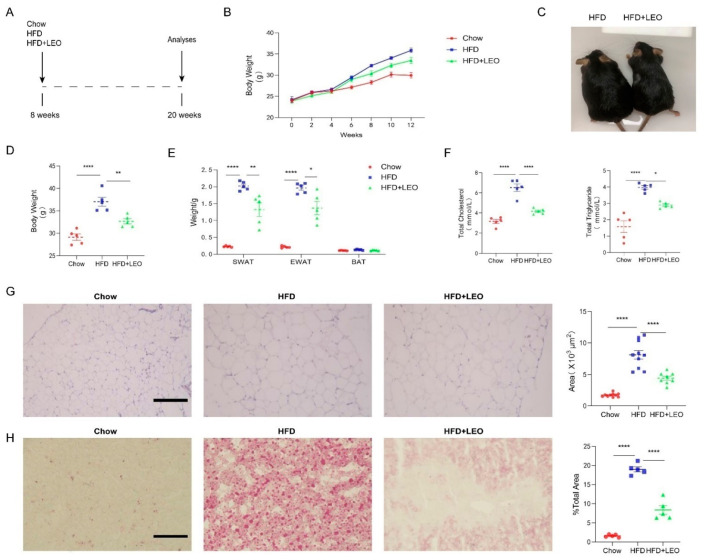
LEO alleviates obesity and lipid deposition. (**A**) Experimental design and grouping. (**B**) Body weight of mice at the start and at 2, 4, 6, 8, 10, and 12 weeks after a normal diet (chow), a high-fat diet (HFD), or a high-fat diet with LEO (HFD + LEO). (**C**) Representative image of mice body. (**D**) Body weight of mice fed chow, HFD, or HFD + LEO after 12 weeks. (**E**) SWAT, EWAT, and BAT weight after 12 weeks. (**F**) The levels of total cholesterol and total triglycerides were measured, and statistical analysis was conducted. (**G**) Representative pictures of EWAT stained by H&E, scale bar = 100 µm. Quantitative analysis of the adipocyte area. (**H**) Representative pictures of liver stained by oil red, scale bar = 100 µm. Quantitative analysis of percentage of lipid droplet area. Data are shown as means ± S.E.M. *n* = 5 per group. * *p* < 0.05, ** *p* < 0.01 and **** *p* < 0.0001 between groups.

To investigate the effect of LEO on body weight and blood lipid, C57BL/6J mice were exposed to a high-fat diet for 12 weeks with or without LEO (Figure 1A). LEO or normal saline was intragastrically administered once per day to the obese mice for 3 months. The body weight of mice in the HFD group was recorded weekly (Figure 1B). The weight of mice treated with LEO was less than PBS treated in HFD mice after 3 months (Figure 1C,D). As shown in Figure 1E, LEO ameliorated the weight of SWAT and EWAT induced by HFD. Obesity is associated with high-serum cholesterol (TC) and TG. The results showed that LEO significantly decreased the serum cholesterol (TC) and TG (Figure 1F). H&E staining in Figure 1G revealed that LEO ameliorated the adipocyte hypertrophy of the adipose tissue in obese mice. The oil red O staining of the liver confirmed that mice fed high-fat diethad more fat deposition than mice fed normal food and LEO dramatically reduced the fat deposition caused by obesity (Figure 1H). These results indicated that LEO reduced the weight of obese mice and prevented the fat deposition in the liver caused by obesity.

### 3.2. LEO Improves Inflammation In Vivo and In Vitro

**Figure 2:**
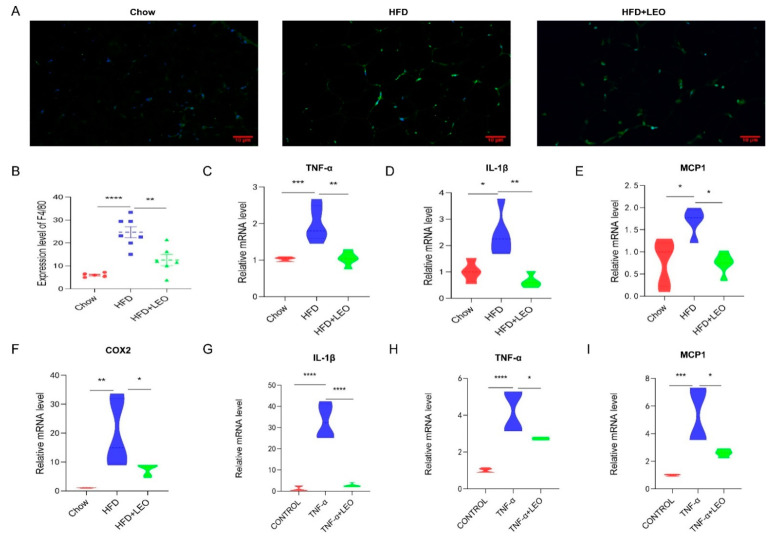
LEO improves inflammation. (**A**) Representative immunofluorescence pictures of the expression and nuclear translocation of F4/80 in three groups’ EWAT. (**B**) Quantitative analysis of the ratios of F4/80. (**C**–**F**) mRNA expression of TNF-α, IL-1β, MCP-1, and COX-2 in the EWAT; *n* = 5 mice per group. (**G**–**I**) mRNA expression of TNF-α, IL-1β, and MCP-1 in the RAW264.7 cells treated with 100 ng/mL of TNF-α with or without 20 μM of LEO for 8 h. Data are shown as means ± S.E.M. * *p* < 0.05, ** *p* < 0.01, *** *p* < 0.001, and **** *p* < 0.0001 between groups.

Obesity induces low-grade chronic inflammation, which refers to multiple organs, including the adipose tissue, liver, and heart [11]. LEO alleviated macrophage infiltration in adipose tissue (Figure 2A). To further elucidate the effect of LEO on inflammation in adipose tissue, we examined the expression levels of the proinflammatory factor, including TNF-α, IL-1β (interleukin-1β), MCP-1 (monocyte chemotactic protein-1), and COX2. As is shown in Figure 2B–F, LEO inhibited the TNF-α, L-1β, MCP-1, and COX2 expression caused by obesity. Then, we investigated the anti-inflammatory effect of LEO in in vitro experiments. We found that LEO could abolish the increase of inflammatory cytokines mediated by TNF-α (Figure 2G–I). Overall, these results demonstrate the anti-inflammation properties of LEO.

### 3.3. LEO Alleviates Oxidative Stress In Vivo and In Vitro 

**Figure 3:**
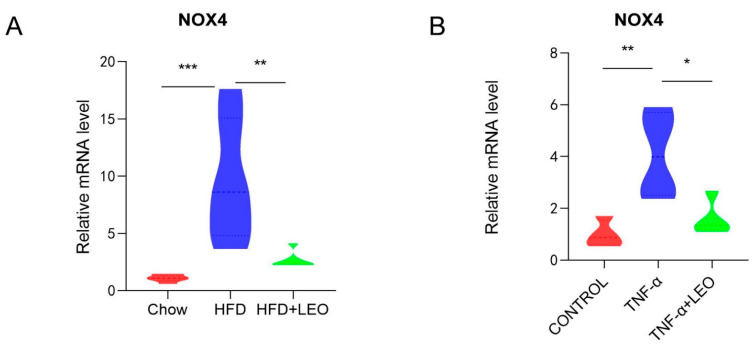
LEO alleviates oxidative stress. (**A**) mRNA expression level of NOX4 in the EWAT; *n* = 5 mice per group. (**B**) mRNA expression level of NOX4 in the RAW264.7 cells. Data are shown as means ± S.E.M. * *p* < 0.05, ** *p* < 0.01, *** *p* < 0.001 between groups.

Studies have confirmed that LEO can decrease ROS formation and block the activation of NOX4 and NF-κB in cardiac fibroblasts to inhibit the cardiac fibrosis [17]. NOX4, the only subtype of the NOX family to produce H_2_O_2_, has the highest renal expression level and a wider range of biological functions than other NOX family subtypes [18]. In order to investigate whether LEO alleviated the oxidative stress induced by obesity, NOX4 mRNA levels were measured both in vivo and in vitro. We demonstrated that LEO could inhibit NOX4 mRNA levels in the adipose tissue of obese mice (Figure 3A). After treating macrophages with TNF-α, NOX4 mRNA levels were increased, but NOX4 levels were significantly inhibited after LEO treatment (Figure 3B). These data indicated that LEO inhibited the oxidative stress induced by obesity.

### 3.4. LEO Ameliorates Mesenteric Artery Dysfunction Induced by Obesity 

**Figure 4:**
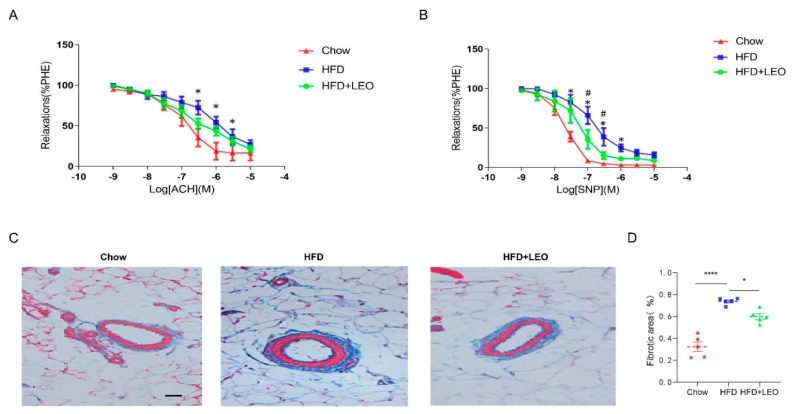
LEO improves vascular function and remodeling. (**A**) Endothelium-dependent relaxation (EDR) of mesenteric artery ring from groups of mice in response to ACH. * *p* < 0.05 between chow group and HFD group. (**B**) SNP-induced endothelium-independent relaxation of mesenteric artery ring from groups of mice. * *p* < 0.05 between chow group and HFD group. ^#^ *p* < 0.05 between HFD group and HFD + LEO group. (**C**) The structure of collagen fiber was observed by Masson’s trichrome staining. Collagen fibers are in blue and the smooth muscle fibers are in red; *n* = 5 mice per group. (**D**) The quantitative analysis of the percentages of collagen content. Data are shown as means ± S.E.M. * *p* < 0.05 and **** *p* < 0.0001 between groups.

In order to understand the effect of LEO on vascular diastolic function, we conducted the vascular ring experiment. Sodium Nitroprusside (SNP) is a powerful vasodilator subordinate to the nitro vasodilator and can directly relax the vascular smooth muscle of the small arteries and veins [19]. SNP is the direct donor of NO, which has the powerful function of relaxing the vascular smooth muscle as well as anti-inflammatory and anti-platelet aggregation [20]. ACH (acetylcholine) promotes the release of NO from vascular endothelial cells, which leads to the relaxation of adjacent smooth muscle cells [21]. Therefore, ACH is an endothelium-dependent vasodilator while SNP is non-endothelium-dependent. We proved that SNP and ACH were able to dilate the mesenteric arteries of the three groups of mice pre-constricted by PE. Obese mice had a decreased ability to dilate their mesenteric arteries compared to the chow group (Figure 4A). The mesenteric artery dilation response of obese mice treated with LEO increased and the difference was statistically significant when the SNP concentration was 1 × 10^−7^, 3 × 10^−7^ mol/L (*p* < 0.05). However, when the vasodilator was ACH, there was no statistical difference in improving the vasodilation ability of LEO (Figure 4B). These results suggest that LEO prefers to improve the vascular tone in a non-endothelium-dependent manner. To discuss the effect of LEO on vascular remodeling, we observed the degree of mesenteric artery fibrosis by Masson’s trichrome staining. We found that LEO significantly improved vascular fibrosis in obese mice (Figure 4C,D). All the above results proved the effect of LEO on vascular remodeling.

### 3.5. LEO Binds YTHDF1 Protein

**Figure 5:**
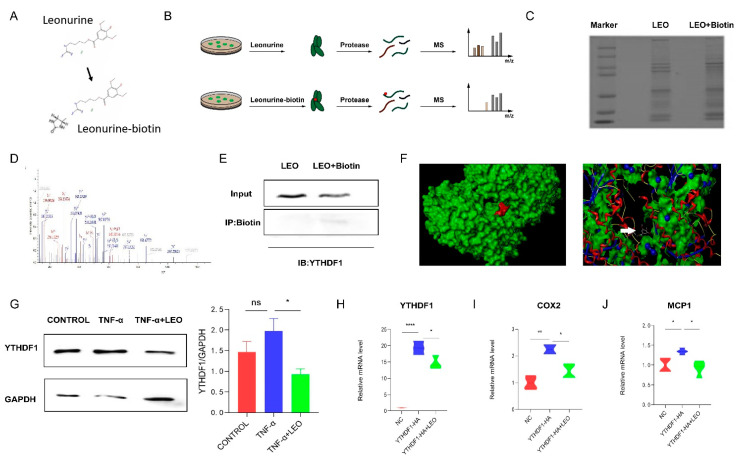
LEO targets YTHDF1 protein. (**A**) The chemical constructions of LEO and the biotin-conjugated LEO. (**B**) Flowchart of LIP-MS analysis. Cells were incubated with LEO or biotin-conjugated LEO for 6 h. Then, proteins pulled down by streptavidin beads were used for LC-MS analysis. (**C**) The image of proteins pulled down by streptavidin beads by using sodium dodecyl sulfate polyacrylamide gel electrophoresis. (**D**) Mass spectrum peak of YTHDF1 by LC-MS analysis. (**E**) RAW264.7 cells were incubated with LEO-biotin or LEO, followed by pull-down assay by using streptavidin beads; representative blot from three independent experiments is shown. (**F**) Representative images of autodocking for LEO and YTHDF1. (**G**) The protein level of YTHDF1 and GAPDH were detected by western blot in cell lysates of TNF-α (100 ng/mL)-stimulated RAW264.7 cells, which were pretreated with or without LEO. Image J was used to analyze gray value. Representative blot from three independent experiments is shown. (**H**) RAW264.7 cells were transfected to express YTHDF1-HA and mRNA expression levels of YTHDF1 were measured by qPCR. (**I**,**J**) RAW264.7 cells were transfected to express YTHDF1-HA and mRNA expression level of COX2 and MCP-1 was measured by qPCR. Data are shown as means ± S.E.M. * *p* < 0.05, ** *p* < 0.01 and **** *p* < 0.0001 between groups.

To identify potential targets of LEO, we employed LC-MS to identify cellular proteins that could directly bind LEO, and LEO was conjugated with biotin (Figure 5A). Then, cell lysates were incubated with LEO-biotin or LEO, followed by the pull-down assay using streptavidin beads. LC-MS was performed on the protein that was pulled down by streptavidin beads (Figure 5B,C). By comparing the contents of proteins pulled down by LEO-biotin, we focused on YTHDF1. The identification of YTHDF1 by LC-MS is shown in Figure 5D. We next confirmed that LEO-biotin could pull down YTHDF1 in RAW264.7 (Figure 5E). We applied the Surflex-Dock software to predict the autodocking of LEO and YTHDF1, and total score was 5.6234 (Figure 5F). Western blotting also showed that the expression of YTHDF1 increased under RAW264.7 inflammation but decreased after LEO treatment (Figure 5G). Next, we overexpressed YTHDF1 in RAW264.7 cells and observed that the mRNA expression of YTHDF1 was decreased by LEO treatment (Figure 5H) [22]. Considering that YTHDF1 knockout reduced intestinal damage and inflammatory factor expression during endotoxin shock, we examined the expression of inflammatory factors after overexpressingYTHDF1. As is shown in Figure 5I, overexpression of YTHDF1 resulted in the increased mRNA levels of inflammatory factors and LEO suppressed the inflammation caused by YTHDF1.

## 4. Discussion

**Figure 6:**
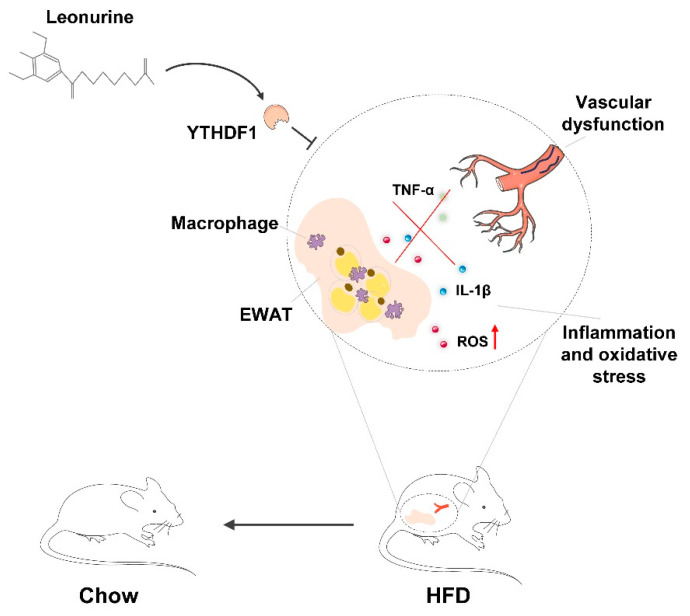
Schematic diagram of the mechanism of LEO alleviating oxidative stress in adipose tissue and vascular dysfunction of obese mice.

The present study revealed that LEO has a positive effect on protecting against vascular dysfunction during obesity-related metabolic syndrome. LEO prevents the progression of obesity by mitigating HFD-induced weight gain, adipocyte hypertrophy, and lipidosis. LEO attenuates oxidative stress and inflammation especially in EWAT and vascular dysfunction. In addition, we found that LEO directly targets YTHDF1 and LEO treatment inhibits YTHDF1-induced inflammation and oxidative stress (Figure 6).

A high-fat diet is one of the important causes of obesity [23,24]. As expected, the body weight of mice fed a high-fat diet was significantly higher than that of those fed a normal diet. Adipose tissue dysfunction in obese mice also includes adipocyte hypertrophy, adipocyte hyperplasia, and hyperlipemia [25,26], which is supported by our results. As was seen in our study, the body weight, the adipose tissue weight, and the area of adipocyte in therapeutic mice, which were given intragastric administration of LEO, were reduced. LEO improved the lipid profile of arteriosclerosis by reducing total cholesterol, total triglyceride, and LDL-C, but did not affect HDL-C levels in plasma [27,28]. Our data also demonstrated the lowering effect of LEO on plasma total cholesterol and total triglyceride in obese mice. 

Obesity and metabolic disorders are accompanied by chronic low-grade inflammation. Inflammatory cytokines in adipose tissue induced by obesity, such as TNF-α, IL-1β, and IL-6, increased [29,30,31]. In addition, adipose tissue macrophages in obese mice showed more pro-inflammatory phenotypes. LEO attenuated inflammatory responses, which was supported by decreasing TNF-α and IL-1β production in LPS-treated microglia, inhibiting COX-2 mRNA and protein expression in LPS-induced mouse mastitis [32,33,34]. Here, we demonstrated that LEO down-regulated TNF-α, IL-1β, MCP-1, and COX2 production in the adipose tissue of obese mice. Similarly, elevated TNF-α, IL-1β, and MCP-1 was seen in RAW264.7 cells stimulated by TNF-α, indicating the generation of inflammation. These data suggest that the down-regulation of TNF-α, IL-1β, MCP-1, and COX2 by LEO decreases the inflammation of adipose tissue macrophages.

Increased oxidative stress in accumulated fat is an important pathogenic mechanism of obesity-associated metabolic syndrome [35,36,37]. ROS production was selectively increased in the adipose tissue of obese mice, accompanied by increased NADPH oxidase expression and decreased antioxidant oxidase expression [38,39]. The NADPH oxidase NOX4 is a hydrogen peroxide (H_2_O_2_)-producing enzyme [40]. NOD1 (nucleotide-binding oligomerization domain protein-1) activation provokes oxidative stress in adipocytes via NOX1/4, which contributes to the induction of inflammatory response [41]. Our results showed that LEO decreased NOX4 production in obese mice compared with normal mice. We also observed in vitro that the expression of NOX4 in TNF-α–challenged RAW264.7 was higher than that in TNF-α + LEO-challenged RAW264.7 cells. Given the inhibitory effect of LEO on NOX4, we conclude that LEO can attenuate oxidative stress by regulating the balance between reactive oxygen species.

Substantial evidence suggested that oxidative stress is involved in vascular injury and thrombus formation [42]. Oxidative damage is crucial in both vascular endothelial cell injury and insufficient angiogenesis in the process of tissue repair, which may lead to aggravating thrombosis [43]. Dysregulated adipocytokines and adipokines in obesity, such as leptin, resistin, TNF, and IL-6, are associated with the vascular dysfunction process [44,45]. We demonstrated that the obese mice showed increased vascular dysfunction and fibrosis, which may be associated with increased levels of inflammatory factors, such as TNF-α and IL-1β, and oxidative stress factors in adipose tissue. LEO participates in the occurrence and development of vascular diseases through anti-inflammatory and antioxidant stress [46]. Our study also confirmed that LEO exerted a protective effect on the vascular remodeling in obese mice. The degree of vascular fibrosis in LEO-treated mice was reduced compared with Chow mice. We used endothelium-dependent vasodilator ACH and endothelium-independent vasodilator SNP in a vascular reactivity test. ACH mediates a transient elevation of free calcium in endothelial cells, which triggers the synthesis of nitric oxide (NO), the endothelium-derived hyperpolarizing factor (EDHF), and eicosanoids derived from arachidonic acid (AA) [47]. SNP increases guanosine 3′5′-monophosphate via the release of nitric oxide (NO), resulting in relaxation of the vascular smooth muscle [48]. We found LEO ameliorated obesity-induced diastolic dysfunction when the vasodilator was SNP but not when the vasodilator was ACH. This is probably because ROS can increase contraction by quenching the bioavailability of NO, depolarize vascular smooth muscle cells by inhibiting potassium channels, and induce calcium sensitization [49]. Given the effects of LEO on ROS production, we can predict that LEO improves smooth muscle cell function by reducing ROS production.

Finding the direct target of LEO is of great significance to further explore its pharmacological mechanism and clinical application, but the target is still unclear. We designed biotin-conjugated LEO and added it in cell culture medium for 6 h, and then incubated it with streptavidin beads. We found that YTHDF1 may be the target by analyzing the result of LC-MS and verified it by the pull-down experiment. During endotoxic shock, YTHDF1 knockout down-regulated intestinal damage and cytokines [22]. NLRP3 transcripts were the targets of YTHDF1 to regulate inflammatory signaling and apoptotic pathways, which further promoted inflammatory processes and oxidative stress. Through our experiments, we observed that LEO can inhibit the increase of pro-inflammatory factors caused by YTHDF1 overexpression, which was consistent with previous results.

These findings support the distinct role of LEO in oxidative stress and inflammation, which further demonstrates that LEO can inhibit obesity, lipid deposition, and microvascular fibrosis. Given the pivotal role of oxidative stress in cardiovascular disease and other metabolic disorders, our study indicates the potential application value of LEO as a new therapeutic drug against metabolic syndrome caused by obesity.

## 5. Conclusions

In conclusion, we showed that LEO attenuates obesity-induced adipose tissue inflammation, oxidative stress, and vascular remodeling. In addition, we demonstrated that the target of LEO is YTHDF1, which has been shown to play an important role in inflammatory response. Thus, the present study confirmed the potential clinical utility of LEO for the treatment of obesity and related metabolic disorders.

## Figures and Tables

**Table 1:** List of mouse primers used for RT-PCR.

Gene Name	Sequence (5′–3′)
GAPDH-F	CTAAAGGGCATCCTGGGC
GAPDH-R	TTACTCCTTGGAGGCCAT
MCP-1-F	CAGGTCCCTGTCATGCTTCT
MCP-1-R	GTGGGGCGTTAACTGCATCT
IL-1β-F	GCAACTGTTCCTGAACTCAACT
IL-1β-R	ATCTTTTGGGGTCCGTCAACT
TNF-α-F	CCCTCACACTCAGATCATCTTCT
TNF-α-R	GCTACGACGTGGGCTACAG
NOX4-F	CCAAATGTTGGGCGATTGTGT
NOX4-R	ATCCATACTCTGCTGTGCCA
YTHDF1-F	CTGCAGTTAAGACGGTGGGT
YTHDF1-R	TAGCAATGGCTGCCCATGAA

## Data Availability

Data is contained within the article.

## Supplementary Materials

The following supporting information can be downloaded at: https://www.mdpi.com/article/10.3390/antiox11071338/s1, Table S1: Key resources table.
