# Supplementary material for: Leonurine Attenuates Obesity-Related Vascular Dysfunction and Inflammation
Source: Antioxidants (Basel). 2022 Jul 8;11(7):1338. doi: 10.3390/antiox11071338 (PMC9311755; doi:10.3390/antiox11071338)
Supplement: Supplementary file 1 [file antioxidants-11-01338-s001.zip › antioxidants-1770039-supplementary.pdf]

Table S1. Key resources table.

REAGENT or RESOURCE	SOURCE	IDENTIFIER
YTHDF1 polyclonal antibody	Proteintech	Cat# 17479-1-AP
GAPDH polyclonal antibody	Proteintech	Cat# 10494-1-AP
Anti-F4/80 antibody	ABCAM	Cat# ab16911
Goat anti-rat IgG(H+L) highly cross-absorbed secondary antibody, Alexa Fluor™ Plus 488	Invitrogen	Cat# A21206
HRP-conjugated affinipure goat anti-mouse IgG(H+L)	Proteintech	Cat# SA00001-1
HRP-conjugated affinipure goat anti-rabbit IgG(H+L)	Proteintech	Cat# SA00001-2
leonurine	Selleck	Cat# S389001
SYBR Green Master Mix	EZBioscience	Cat# A0012
Reverse Transcription Master Mix	EZBioscience	Cat# A0010G
RNA Purification Kit	EZBioscience	Cat# B2551
Oil red dye	Servicebio	Cat# GP1067
Hematoxylin and eosin kit	Servicebio	Cat# GP1031
Masson trichrome kit	Servicebio	Cat# G1006
Lycerin gelatin seal tablets	Servicebio	Cat# G1402
C57BL/6J	Charles River	
DAPI	Biosharp	Cat# BS097
